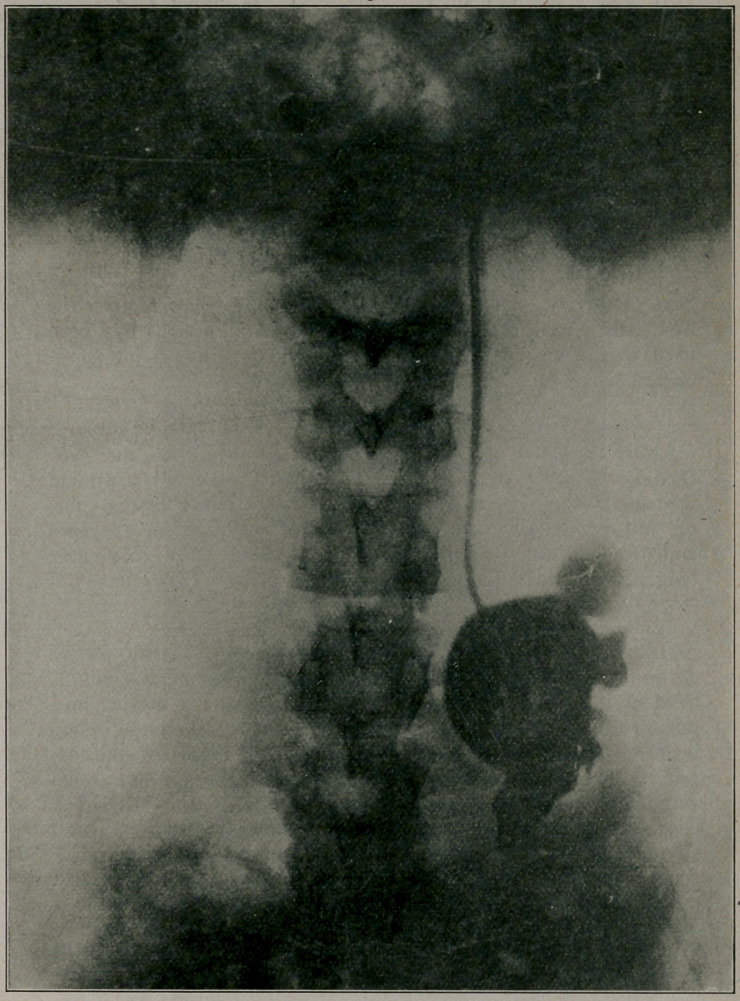# Pyelography (Radiography of Ureter and Renal Pelvis) in Diagnosis of Surgical Conditions of Kidney

**Published:** 1913-02

**Authors:** 


Pyelography (Radiography of Ureter and Renal Pelvis) in Diagnosis of Surgical Conditions of Kidney.—George A. Matteson of Providence, in *Providence Med. Jour.*, November, 1912. Several interesting cases are reported. The name "pyelography" was suggested by Voelcker of Heidelberg, who did the first work of the sort in 1906. Braasch of Rochester, Minn., is mentioned as having done the most extensive work in America. The technic consists in emptying the bowel, cystoscopy under local anaesthesia by alypin, ureteral catheterization, injection of 10-15 per cent. collargol, radiography. If an ounce can be injected without causing pain, it is presumptive evidence of hydronephrosis. Three cases were similar to the one illustrated. In another, hydronephrosis was shown by the shadow, in addition to the denser shadow of a calculus previously noted but now demonstrated to be within the dilated renal pelvis. In another case, the diagnosis of movable kidney was verified and hydronephrosis was excluded. In another, colon bacillus infection was shown not to be accompanied by hydronephrosis, nephroptosis, etc. In two others negative results excluded the kidney as the cause of vague right sided pains.

## Figures and Tables

**Figure f1:**
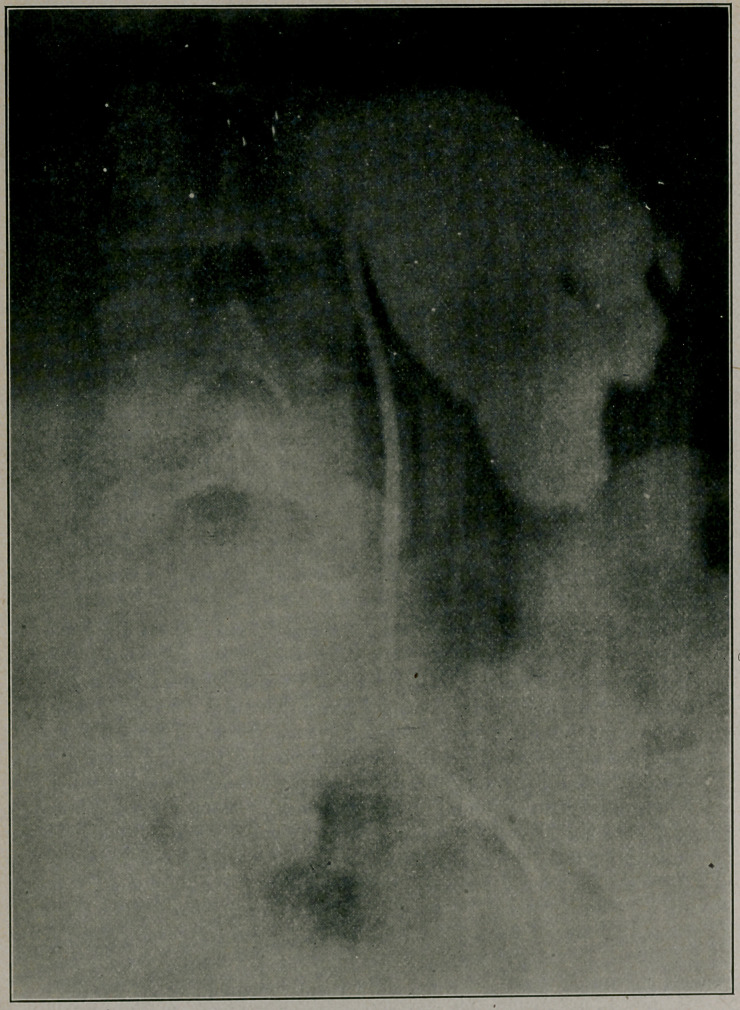


**Figure f2:**